# Surface-Enhanced Raman Scattering (SERS) Spectroscopy for Sensing and Characterization of Exosomes in Cancer Diagnosis

**DOI:** 10.3390/cancers13092179

**Published:** 2021-04-30

**Authors:** Luca Guerrini, Eduardo Garcia-Rico, Ana O’Loghlen, Vincenzo Giannini, Ramon A. Alvarez-Puebla

**Affiliations:** 1 Department of Physical and Inorganic Chemistry, Universitat Rovira i Virgili, Carrer de Marcel·li Domingo s/n, 43007 Tarragona, Spain; 2 Fundación de Investigación HM Hospitales, San Bernardo 101, 28015 Madrid, Spain; egarcia@hmhospitales.com; 3 School of Medicine, San Pablo CEU, Calle Julian Romea, 18, 28003 Madrid, Spain; 4 Epigenetics & Cellular Senescence Group, Blizard Institute, Barts and The London School of Medicine and Dentistry, Queen Mary University of London, London E1 2AT, UK; a.ologhlen@qmul.ac.uk; 5 Instituto de Estructura de la Materia (IEM-CSIC), Consejo Superior de Investigaciones Científicas, 28006 Madrid, Spain; v.giannini@csic.es; 6 Technology Innovation Institute, Masdar City, Abu Dhabi 9639, United Arab Emirates; 7 ICREA, Passeig Lluis Companys 23, 08010 Barcelona, Spain

**Keywords:** exosomes, cancer diagnosis, sensing, early detection, plasmonics, nanoparticles, surface-enhanced Raman spectroscopy

## Abstract

**Simple Summary:**

The distinct molecular and biological properties of exosomes, together with their abundance and stability, make them an ideal target in liquid biopsies for early diagnosis and disease monitoring. On the other hand, in recent years, nanomaterial-based optical biosensors have been extensively investigated as novel, rapid and sensitive tools for exosome detection and discrimination. The scope of this review is to summarize and coherently discussed the diverse applications, challenges and limitations of nanosensors based on surface-enhanced Raman spectroscopy (SERS) as the optosensing technique.

**Abstract:**

Exosomes are emerging as one of the most intriguing cancer biomarkers in modern oncology for early cancer diagnosis, prognosis and treatment monitoring. Concurrently, several nanoplasmonic methods have been applied and developed to tackle the challenging task of enabling the rapid, sensitive, affordable analysis of exosomes. In this review, we specifically focus our attention on the application of plasmonic devices exploiting surface-enhanced Raman spectroscopy (SERS) as the optosensing technique for the structural interrogation and characterization of the heterogeneous nature of exosomes. We summarized the current state-of-art of this field while illustrating the main strategic approaches and discuss their advantages and limitations.

## 1. Introduction

In the impending decades, cancer is set to become a major cause of morbidity and mortality across all regions of the globe [1], with an estimated 13.2 million related deaths by 2030 [1,2]. Thus, the development of more effective treatments and, fundamentally, new forms of prevention and early diagnosis are both necessary strategies to achieve a cure [3]. In fact, diagnosis at the very earliest stages improves cancer outcomes by prompting treatments aimed at preventing the disease development to incurable stages.

The prevailing theory about the origin of cancer indicates that a primary tumor develops for a long time, from a subclinical or microscopic level, before it spreads at distance (metastasis) [4]. To be clinically detectable, a tumor must reach a size of ca. 1 cm^3^, which approximatively contain 10^9^ cells [5]. Therefore, at the time of diagnosis, there is a high probability of prior dissemination. As a result, there is an urgent need for new technologies capable of detecting the presence of tumor cells before the disease emerges as clinically visible. In this regard, exosome-based liquid biopsy in peripheral blood and other body fluids is among the most promising techniques for pre-metastatic cancer diagnosis [6]. The validity of such an approach builds upon the current concept and understanding of metastasis [7,8,9,10,11]. Indeed, it has been recognized that before they spread to distant sites, the original or primary tumors “communicate” with cells and tissues of other organs, as well as their surrounding environment, to prepare what will be eventually a metastatic niche. This horizontal intercellular communication takes place through exosomes. In bone-marrow [7], for example, hematopoietic progenitor cells that express VEGFR1 are located in tumor premetastatic sites and form cellular clusters induced by exosomes originated in primary tumor cells. In these niches, such cells express VLA4 and certain integrins that facilitate the arrival of tumor cells, a process that is also mediated by exosomes [12]. On the other hand, by this communication, tumor stem cell deference can be induced from normal cells.

Exosomes were first described in the 1960s as vesicles related to coagulation processes derived from platelets and, two decades later, they were associated with enzymatic functions [13]. Subsequent observations showed that these vesicles were generated as cell desquamation in the reticulocyte maturation process [14]. Exosomes are small, single-membrane vesicles approximately between 30 and 150 nm diameter, secreted by practically all cells into the extracellular environment through the fusion of specific endosomes (multivesicular bodies, MVBs) with the plasma membrane. MVBs are formed by primary endosomes which are incorporated as “intraluminal vesicles” (ILV) via inward budding of the multivesicular body membrane [12]. They can follow this secretory pathway towards the extracellular environment or a degradative pathway through their fusion with lysosomes. Exosomes have been shown to intervene in multiple functions (e.g., immune response, healing, viral synthesis, antigenic presentation, etc.) [15,16]. In cancer, multiple functions have been attributed to exosome-mediated communication such as reprogramming of stromal cells, initiation of metastasis, preparation of metastatic niches, modelling of the immune response and extracellular matrix, drug resistance, antigen presentation, etc. [17]. Notably, such intercellular exosomal communication takes place in both directions: from tumor cell to normal cell and vice versa. Thus, tumor cells can gain capacities such as “invasiveness” or enhance their proliferative efficiency. An example of this reverse communication process has been observed for normal adipocytes, which secrete exosomes carrying proteins involved in the oxidation of fatty acids that are eventually incorporated into melanoma cells. Such process culminates in an increase of this function in malignant cells, which enhances their migration and invasion capabilities [18].

**Figure 1:**
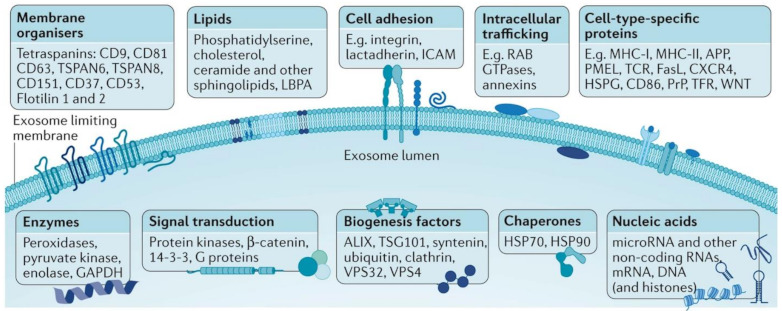
General outlook of the exosome membrane composition and different molecular cargoes in the lumen which can markedly vary based on the parental cell and vesicle biogenesis. Adapted with permission from [33]. Copyright 2018, Nature Publishing.

Exosome lumen and membrane carry biological and genetic information related to their parental cell types as they are selectively enriched of specific nucleic acids (e.g., mRNA, miRNA, tRNA, etc.), proteins (e.g., integrins, immunoglobulins, growth factors, cytoskeletal protein actin and tubulin, endosomal sorting complex required for transport ESCRT-related proteins, hsp90, hsp70, tetraspanin, major histocompatibility complex), lipids, metabolites and glycoconjugates (Figure 1) [19,20]. It is also worth stressing that the exosome biogenesis itself can significantly impact their composition and functionality [3]. In this sense, the most significant aspect is that exosome molecular composition is not a mere random replica of the original cell but is selected and specific. The overall mechanisms of such selection (sorting) are very complex and have been only recently being unveiled. In general terms, the integration of the molecular components into endosomes takes place selectively via recognition by specific sequences of nucleotides or peptides. This mechanism is similar to ubiquitination, a process that marks and selects proteins destined for endosomal degradation [21]. In this way, RNA molecules containing a specific sequence (EXOmotif) are recognized by certain proteins (hnRNPA2B1) that facilitate their entry into exosomes. These proteins also undergo an activation process through reactions known as sumoylation, which helps integration, or isegilation (incorporation of ISG15 into TSG101), which inhibits the generation of exosomes (or facilitates their elimination by fusion to lysosomes) [22,23]. As a result, characteristic miRNAs in exosomes nucleic acid cargoes, while not expressed in the corresponding healthy tissues, have been detected, for instance, in breast cancer [24] and lung adenocarcinoma [25], demonstrating their validity as unique disease markers. Similarly, exosome membrane protein composition has also shown to be correlated with the nature of the originating cell and the transformation events that have undergone [26,27], which also make them promising diagnostic biomarkers and therapeutic targets [26,27,28]. This is consistent with the central role played by surface membrane proteins of exosomes in malignant processes such as metastasis [29,30]. Moreover, glycans bound to surface proteins and outer lipids are also found onto the exosomal surfaces [19], and they have been reported to play an important biological role, among others, in the exosome uptake [31]. Overall, the unique distinct molecular and biological properties of exosomes, together with their abundance and stability, make them an ideal target in liquid biopsies not only for early diagnosis [6] but also for disease monitoring and, finally, an opportunity for cancer cure [32].

## 2. Isolation and Characterization of Exosomes

The pronounced molecular and size heterogeneity of exosomes, even for vesicles originating from the same parental cells, confers a central role to isolation methods in (i) separating exosomes from potentially interfering protein aggregates, lipoparticles, viruses and cell debris in cell culture supernatants or bodily fluids, and (ii) discriminating different exosome subpopulations that could be related with different pathological states and stages of disease progression [33]. The gold standard for exosome isolation is differential centrifugation which, through several centrifugation rounds (such as ultra-high-speed centrifugation or ultracentrifugation), selectively precipitates the vesicles of interest with high purity [34]. Ultracentrifugation, however, is a slow separation method with low recovery efficiency (<25%) that requires costly and bulky instrumentations and, thus, is not suitable for the point-of-care diagnosis [35]. Additional separation strategies, exploiting diverse physiochemical properties of exosomes, include size exclusion chromatography, ultrafiltration, immunoaffinity capturing, charge neutralization-based polymer precipitation, and microfluidic techniques, each of them with a characteristic set of advantages and disadvantages [35]. Physical characterization of the isolated vesicles (i.e., enumeration, size distribution and morphology) are commonly determined via nanoparticle tracking analysis (NTA), flow cytometry, microscopy methods (e.g., transmission electron microscopy, TEM; scanning electron microscopy, SEM) and dynamic light scattering [36,37,38]. On the other hand, exosome protein quantification is conventionally carried out via Western blotting and enzyme-linked immunosorbent assay (ELISA) [38]. However, western blotting typically requires complex and time-consuming procedures as well as relatively large volumes of biosamples; whereas ELISA fails to execute multiplexed analysis. Differently, nucleic acid cargoes, mainly RNAs, are commonly analyzed upon extraction via amplification and sequencing techniques (e.g., PCR, next-generation sequencing) [38].

**Figure 2:**
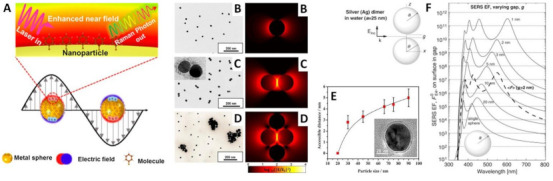
(**A**) Outline of the surface-enhanced Raman scattering effect due to the excitation of localized surface plasmon resonances at a gold nanosphere/air interface. Reprinted with permission from [59]. Copyright 2017, American Chemical Society. (**B**–**D**) TEM images and calculated electrical fields for an isolated silver nanoparticle of 45 nm diameter and its corresponding dimer and tetramer (as a representative example of a largercluster), respectively, under a 514 nm excitation laser (interparticle gap, g = 1.31 nm). Reprinted with permission from [58]. Copyright 2015, American Chemical Society. (**E**) TEM image of a silica-coated silver nanoparticle and plot of the maximum silica shell thickness at which the SERS signal of rhodamine 6G is still detectable (i.e., accessible distance) as a function of nanoparticle diameter. Adapted with permission from [53]. Copyright 2015, American Chemical Society. (**F**) Dimer composed of two identical silver nanospheres (radii *a* = 25 nm) separated by a gap *g* (the incoming wave is polarized along the axis of the dimer) and their theoretical SERS enhancement factors calculated at the point on the surface in the gap (i.e., hot-spot) as a function of the excitation wavelength for different gaps. The thick dashed line is the average SERS enhancement factor in the case of a 2 nm gap. Adapted with permission from [51]. Copyright 2009, Elsevier.

In recent years, nanomaterial-based optical biosensors have been extensively investigated as novel, rapid and sensitive tools for exosome detection and discrimination [39,40,41]. Within the field of nanoplasmonic, surface-enhanced Raman spectroscopy (SERS) has emerged as a powerful optical technique for a very broad range of applications [42,43,44,45], with the most intriguing one being in biosensing and clinical diagnostic [46,47,48,49,50]. SERS is an analytical technique that relies on the excitation of strong electromagnetic fields (i.e., localized surface plasmon resonances, LSPRs) at the surface of plasmonic materials (mainly, silver and gold nanostructures) (Figure 2A). As a result of the excitation of the molecular species with the LSPR rather than with the illuminating light, the Raman scattering of molecules located in close contact or directly attached to the plasmonic substrate undergoes a notable amplification, up to a factor of ca. 10^10^–10^11^ [51]. Thus, SERS simultaneously affords an ultra-sensitive optical response based on the plasmonic associated intensification and the intrinsically rich structural information contained in the Raman spectra. In Figure 2 we provide few illustrative examples with the aim of intuitively emphasize some of the key concepts of SERS spectroscopy which have major implications in the application to exosome analysis, as discussed later in the review. We refer the readers to references [46,51,52] for detailed insights on the theoretical and experimental aspects of SERS and related plasmonic substrates. Firstly, Figure 2B depicts the calculated electromagnetic field around a silver nanosphere of 45 nm diameter, hinting the distance-dependent nature of the SERS phenomenon. In fact, the local field enhancements swiftly drop at an increasing distance, *d*, from the metallic surface (the decay is ∼1/(*a* + *d*)^12^ for a nanosphere of radius *a*) [51]. In an explicative study, Kumari et al. [53] synthesized spherical silver colloids of increasing diameter and coat them with silica shells of progressively larger thicknesses (silica prevents the direct contact between the analyte and the nanoparticle). Results show that the SERS intensity decays exponentially for all nanoparticle size as silica shell thickness is increased. On the other hand, the enhancing properties of silver colloids improve with the nanoparticle size up to ca. 100 nm diameter before dropping due radiation effects that reduce the quality of the LSPRs (i.e., plasmon damping) for larger particles. Accordingly, the distance from the metallic surface up to which the SERS signal of the analyte can be observed (i.e., accessible distance) increases with the nanoparticle size up to a maximum of ca. 5 nm distance for ca. 90 nm size colloids. While nanosphere size plays a role in determining the final enhancing properties [54], the largest optical intensifications are, nonetheless, achieved at the tips of sharp protruding features [55,56] and, even more so, at nanometer-sized gaps between metal nanoparticles (i.e., hot-spots) due to interparticle plasmon coupling [52,57]. This latter effect is plainly visualized in Figure 2B–D, which compares the calculated electromagnetic fields in dimers and larger aggregates with that of their parental isolated nanosphere [58]. Notably, local enhancements at the gaps rapidly rise with the shortening of the interparticle distance (see the case of a silver nanoparticle dimer in Figure 2F). However, this simultaneously occurs at an increasing degree of spatial localization (i.e., higher enhancements extend over a smaller volume around the hotspot for smaller gaps) [51]. These aspects highlight the importance of an appropriate structural design of the plasmonic substrate as well as the successful localization of the target molecule within the volumes where the largest enhancements take place. It is also worth noting that, besides the dominant plasmonic-mediated amplification via an electromagnetic mechanism, additional enhancements can result from electron charge transfers between the surface and the analyte (i.e., chemical mechanisms).

Most studies aimed at correlating the intercellular signalling and pathological responses of exosomes with their composition focused on analyzing their respective nucleic acid cargoes which often mirror the phenotypes of their parental cells [19,28,60,61]. SERS-based detection of RNA cargoes extracted from exosomes contained in blood samples of patients have been reported, for instance, for early detection of pancreatic cancer [62] and lung cancer [63]. In these studies, exosomes were separated from plasma and, subsequently, microRNA cargoes were isolated using available kits yielding miRNA elutes to be analyzed. Thus, the scientific challenges and sensing strategies of these approaches are by and large independent of the biomolecule source and fall within the field of nucleic acids SERS detection [64,65,66,67]. On the other hand, in this review, we will focus on the burgeoning body of work on SERS analysis of whole exosomes, a field of research that has been growing at an extremely fast rate in very recent years. For reasons that will be explained shortly, whole exosome SERS analysis mostly focuses and build upon the diversity in their membrane composition.

Broadly speaking, SERS detection approaches can be classified into two main configurations: direct and indirect schemes. In direct SERS, the signal read-out is provided by the acquisition of the intrinsic SERS spectrum of the analyte, which contains a wealth of structural information representative of the molecular structure and composition. This label-free analysis can be carried out with simple and inexpensive plasmonic materials. However, it is usually restricted to the interrogation of relatively pure samples to prevent competing co-adsorptions of other molecular species that could undermine the correct interpretation and reliability of the final SERS spectrum. Moreover, direct SERS characterization of large biomolecules or supramolecular structures such as exosome poses important challenges in terms of understanding and interpretation of complex and often highly similar vibrational patterns. On the other hand, indirect approaches are designed to monitor the extrinsic SERS signal of molecular labels for detection and quantification of the target species. The most common indirect strategy relies on the use of SERS-encoded nanoparticles (or SERS tags) combined with surface ligands for selective recognition (e.g., antibody, aptamers), as optical probes performing similar functions as fluorescent labels [68]. Although labelled methods typically demand elaborate and extensive fabrications of relatively expensive SERS substrates, they also feature ultrasensitivity, high-throughput screening, multiplexing abilities, robust quantitative response in complex media (e.g., biofluids) and suitability to be integrated into miniaturized devices for automated testing, especially at the point-of-care. For these reasons, they have been largely preferred for biosensing applications over direct approaches. Nonetheless, the current literature survey on exosome SERS analysis shows a larger number of reports based on direct approaches [69,70,71,72,73,74,75,76,77,78,79,80,81,82,83,84,85,86,87,88,89,90,91] than indirect ones [92,93,94,95,96,97,98,99,100,101]. This apparent anomaly may be explained by taking into considerations the intrinsic exosomal heterogeneity that currently burdens the identification of specific disease-related biomarkers for selective separation and labelling of clinically relevant exosome subpopulations [90,99,102]. Thus, the more holistic approach of acquiring the vibrational fingerprint of the whole ensemble of molecular constituents, including known and unknown biomarkers, remains a very valuable and effective sensing strategy as compared to indirect analytical methods that selectively inform about one or few structural features.

## 3. Direct Label-Free SERS Analysis of Exosomes

The acquisition of intense, well-defined and reproducible vibrational spectra is key to use direct SERS for sensing purposes. Overall, several factors determine the final intensification and ultimate spectral profile (i.e., band centers, relative intensities, bandwidths) of the vibrational fingerprint. Among those, we can identify inherent features of each element of the SERS analysis, such as the optical properties of the plasmonic material, the Raman cross-section of the target analyte and the experimental set-up (e.g., laser excitation wavelength) [51]. On the other hand, more intertwined variables play also a central role, such as the extent of analyte surface coverage and the relative spatial localization of the analyte with respect to the metallic surface. As previously discussed, the plasmon-mediated electromagnetic enhancement dramatically declines with the distance from the plasmonic surface. This phenomenon accounts for the observation that SERS spectra are typically dominated by the contributions of the first layer of molecules directly exposed to the metal surface. Furthermore, the adsorption of the molecular entity onto the metallic surface may induce both specific orientations and potential alterations of its Raman polarizability which can severely impact the spectral profile of the SERS signal [51]. Thus, the acquisition of reliable SERS spectra is mostly constrained by the careful control and knowledge of all these parameters.

In the liquid phase, the close contact between the analyte and the plasmonic surface is commonly achieved by exploiting the intrinsic chemical affinity of the molecule for gold or silver surfaces, mainly via the formation of metal-O, metal-N, and metal-S bonds (in the typical order of relative increasing strength) or via electrostatic interactions. Alternatively, molecular adhesion can be forced via physical evaporation of the sample solution onto the plasmonic substrate. In this regard, it is worth stressing that for large biomolecules, such as the exosome components (proteins, nucleic acids, etc.) and even further to micro-entities such as cells, a transition from a hydrated to a dried state often results in major structural alterations that usually increase the intra-sample spectral variability [103,104]. All these considerations also justify the need for pre-isolation steps to extract the target biomolecules from complex biological environments containing a multitude of other molecular species which would otherwise compete for the adsorption onto the metallic surface and, eventually, yield unintelligible SERS spectra.

**Figure 3:**
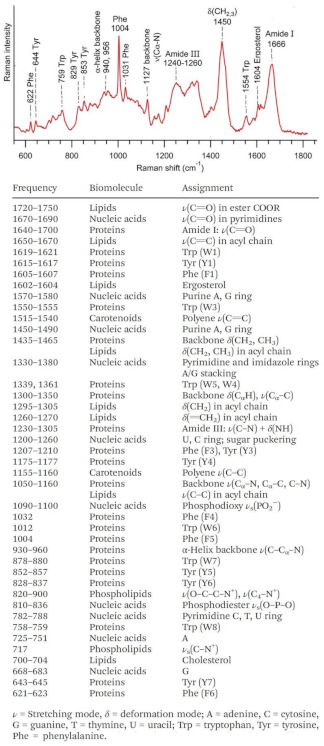
Normal Raman spectrum of exosomes from rat hepatocytes (water buffer contribution was removed via subtraction) and vibrational assignment of the main bands. Adapted with permission from [105]. Copyright 2019, Royal Society of Chemistry.

In the specific case of exosomes analysis, several additional features further increase the complexity of common direct SERS analysis. Firstly, the complex composition of exosomes, which mostly includes a large fraction of biomolecules with typically weak spontaneous Raman scattering (e.g., lipids, proteins), intrinsically generates an intricate vibrational spectral pattern comprising overlapping and often broad features. As a representative example, in Figure 3 we report a normal Raman spectrum of exosomes isolated from rat hepatocytes together with a table illustrating the main vibrational features and their assignment to dominant molecular contributions. As exosomes differentiation occurs via recognition of subtle spectral differences, the use of multivariate mathematic and statistic methods is commonly required for a more accurate spectral analysis. These mathematical methods (e.g., principal component analysis, PCA; partial least square discriminant analysis, PLS-DA, etc.) reduce the high multidimensionality of the large set of vibrational data by identifying a dominant, smaller group of variables that still retains most of the key information of the initial large data set. It is worth noting that the spectral complexity may be also exacerbated by the inherited heterogeneity of the exosome particles, which further stresses the central role of efficient and reliable isolation methods to yield relative pure fractions of exosomes for direct SERS interrogation.

Secondly, the size range itself of exosomes (ca. 30–150 nm) poses additional challenges. In fact, exosomes are large enough to prevent their optimum trapping into nanometric plasmonic gaps (hot spots) capable of concentrating extremely high intense EM fields in the whole analyte volume. Thus, the design and choice of the plasmonic substrate and experimental set-up analysis face two contrasting needs: (i) the necessity to expose exosomes to high electromagnetic enhancements to improve the amplification of their relatively weak Raman scattering; and (ii) the importance to immerse the vesicle in a relatively uniform electromagnetic field so that to minimize heterogeneous enhancements of random portions of exosomes due to different spatial arrangements onto the metallic surface. Indeed, such uneven exposures may lead to high spectral variability even within the same population of exosomes, an outcome that can be further aggravated by the heterogeneous distribution of the diverse molecular components on the exosome surface. Clearly, the reproducibility issue becomes particularly relevant when the SERS spectra are acquired from single/few exosomes rather than large ensembles of particles (i.e., single vs bulk analysis). For instance, Russo et al. [69] observed a significant loss in intra-sample spectral reproducibility, as compared to normal Raman spectroscopy, for drop-cast exosomes on non-uniform plasmonic substrates comprising randomly distributed gold nanostructures. The existence of such a significant number of variables arising from different sources (e.g., origin of the vesicle, isolation protocols, physiochemical characteristics of the plasmonic substrate, sample preparation, experimental set-up, etc.) is most likely the reason why we can observe, from study to study, marked fluctuations of the exosome spectral profiles that appear to go beyond the intrinsic biochemical nature of the interrogated vesicles.

Exosome sizes are, on the other hand, typically too small to facilitate single-particle Raman analysis. This explains why, for instance, single-cell Raman spectroscopy is a well-established and relatively straightforward tool for in vitro and in vivo interrogation of individual living cells while Raman characterization of individual/few exosomes is very limited and requires complex technologies such as exosome trapping via optical tweezers [105,106,107].

Finally, as the electromagnetic enhancement commonly declines very rapidly within few nanometers from the metallic surface, direct SERS analysis of whole exosomes yield spectra that are mostly dominated by the vibrational features of the molecular components of the outer membrane (mainly sugars and proteins). Thus, SERS spectra substantially disregard the lumen content as compared to normal Raman scattering of whole EVs, where nucleic acids contributions are distinguishable in the vibrational pattern [107]. While this aspect prevents the application of SERS as a technique for characterizing the global biomolecular composition of exosomes, it appears not to hamper its viability in diagnostic applications. In this regard, both normal Raman and SERS studies showed that trypsinization of exosomes drastically reduces the capability of differentiating sub-populations of exosomes, including vesicles from diverse cellular sources [73,107]. The enzymatic treatment of exosomes with trypsin promotes the cleavage of most surface membrane proteins and surface glycans, thereby exposing the intraluminal content to plasmonic-mediated signal enhancement. This result highlights the central role of the extraluminal domain for exosome differentiation and, in turn, the validity of the direct SERS approach for whole exosome classification.

**Figure 4:**
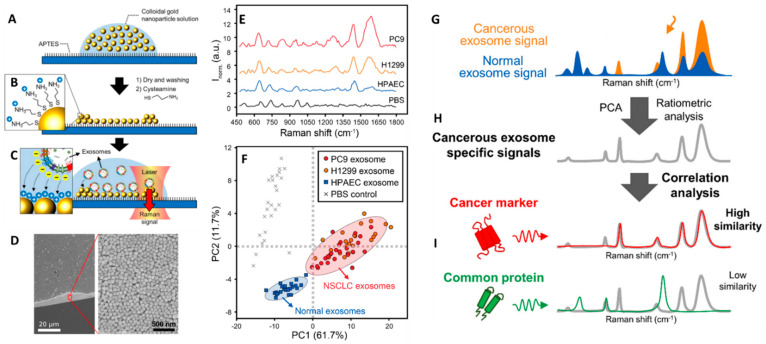
(**A**–**C**) Outline of the substrate fabrication and experimental set-up. (**D**) Scanning electron microscopy (SEM) image at an edge of the substrate. (**E**) SERS spectra of exosomes derived from HPAEC (normal) and H1299, PC9 (lung cancer) cell lines. Phosphate-buffered saline (PBS) was chosen as the experimental control. (**F**) PCA score plot of the SERS data and 90% confidence ellipses. (**G**–**I**) Schematic representation of the experimental process for the identification of unique SERS profile of lung cancer cell-derived exosomes followed by comparison to the profiles of their potential surface protein markers to determine their respective similarity. Adapted with permission from [80]. Copyright 2018, American Chemical Society.

Spherical-like gold and silver colloids synthesized via chemical reduction in solution are easily and reproducibly prepared in large batches at very low cost, yielding very amenable plasmonic materials for SERS analysis of exosomes. Most likely, the simplest and cheapest approach to generate SERS substrates rich in electromagnetic hot-spots is by the direct casting of colloids onto glass slides [79,80]. For instance, Choi and co-workers [80] dried 80 nm gold nanoparticles on a cover glass previously functionalized with 3-aminopropyltriethoxysilane (APTES) to yield a positive surface charge that would promote the adhesion of the negatively charged colloids via electrostatic binding (Figure 4A). Similarly, dried nanoparticle surfaces were further modified with cysteamine to promote the subsequent adsorption of the negatively charged exosomes (Figure 4B). SERS spectra of the vesicles were acquired at the edges of the dried spot (Figure 4C) where very dense nanoparticle clusters accumulate due to the coffee-stain effect (Figure 4D). Figure 4E shows representative SERS spectra of exosomes obtained via size-exclusion column chromatography from HPAEC (normal) and H1299, PC9 (lung cancer) cell lines (phosphate-buffered saline is used as a control). PCA score plot of the SERS data clearly shows the efficient discrimination between normal vs cancer cells-derived exosomes (Figure 4F). Besides a mere differentiation of distinct spectral patterns from normal and cancerous exosomes, the recognition of the molecular origin of the Raman markers at the core of such discrimination would provide a deeper understanding of their biochemical nature and, also, increase the diagnostic and prognostic value of direct SERS analysis. To this end, the authors performed a ratiometric analysis by acquiring the averaged SERS spectra of mixtures of normal and cancerous exosomes at different ratios (the total amount of exosomes was fixed at 10^8^ particles/mL). Subsequently, they identified 13 bands that correlated well with the relative exosome content and which were used as Raman markers for non-small-cell lung carcinoma (NSCLC) derived exosomes (Figure 4G,H). These features were then compared to the vibrational profiles of clinically relevant exosomal protein markers (CD9, CD81, EpCAM, and EGFR). While all these individual protein markers display similar peak compositions, they diverge in relative band intensities thereby generating a unique spectral pattern. Notably, the ensemble of the Raman markers for NSCLC exosomes displayed low similarity for CD9, CD81 and EpCAM spectral fingerprints but high similarity for EGFR, indicating that EGFR expression is a primary variable of NSCLC exosome differentiation (Figure 4I), as further confirmed by immunoblotting analysis.

Besides the direct casting of preformed colloids onto glass-slide surfaces, inexpensive SERS substrate can be generated by in-situ synthesis of plasmonic nanoparticles anchored onto the solid support. In this regard, Ferreira et al. [81] reported the simple fabrication of a hybrid SERS material via in situ silver nanoparticles growth into bacterial cellulose (BC), a low-cost and abundant support obtained from commercial nata de coco. The viability of the substrate for direct SERS analysis of exosomes was demonstrated in the efficient discrimination of exosome samples isolated from MCF-10A (nontumorigenic breast epithelium) and MDA-MB-231 (breast cancer) cell cultures.

**Figure 5:**
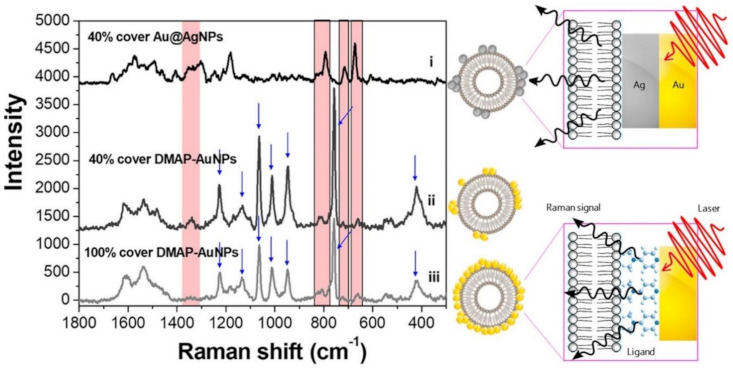
SERS spectra of B16F10 melanoma derived exosomes for 40% coverage with Au@AgNPs or DMAP–AuNPs, and 100% coverage with DMAP–AuNPs. Intense DMAP bands are indicated by blue arrows (these features disappear upon silver coating). An illustrative description of DMAP–AuNPs or Au@AgNPs attached on the exosomes surfaces is also included. Adapted with permission from [88]. Copyright 2019, American Chemical Society.

A practical and straightforward alternative to promote exosome-nanoparticles interactions is by combining vesicles and plasmonic colloids in suspension before their deposition onto a support slide for SERS interrogation [71,79,87,88]. A common drawback of this method is the relatively low affinity of common negatively-charged gold and silver colloids (typically, citrate-stabilized) for similarly negatively-charged exosome membranes that reduces the extent of nanoparticle loading onto the vesicle surface [71]. To tackle this issue, Fraire et al. [88] modified gold nanoparticles with 4-(dimethylamino)pyridine (DMAP) to impart positive charge (DMAP-AuNPs) and, consequently, favor the electrostatic adhesion onto exosomes vesicles derived from B16F10 melanoma cells. In this regard, it is also worth noting that the largest enhancements of the exosome SERS signals have been observed for nanoparticle/exosome ratios yielding approximately 40% coverage, as higher nanoparticle coatings suffer from radiation damping. Regardless, DMAP yields intense bands that markedly overlap with the SERS signal from the vesicle (Figure 5). Such an issue has been circumvented by in situ overgrowing of a sufficiently thick Ag layer on Au nanoparticles (Au@AgNPs) previously attached to the exosomes. The outer metallic coating quenches the DMAP spectral contributions while further boosting the exosome signal by a factor of ca. 5. The acquisition of a “clean” exosome spectrum by removing the interfering DMAP features enabled a more reliable statistical classification of individual exosomes isolated from B16F10 melanoma cells and red blood cells.

Direct interaction of exosomes with traditional gold and silver nanoparticles, either physically forced via evaporation onto a solid support or chemically-mediated in suspension, offers a very simple, inexpensive and straightforward strategy for direct SERS analysis. However, it inherently poses important challenges for obtaining reproducible and uniform SERS responses due to the irregular arrangement of the nanoparticles onto the exosome outer layer. To address these limitations, multiple examples of precisely tailored SERS substrates have been generated profiting from the continuous advances in very diverse areas of nanofabrication technologies [108,109,110]. While each methodology displays a characteristic set of drawbacks and advantages, the fine-tuning of the morphological features of plasmonic materials for maximizing the homogeneity and efficiency of the SERS performances typically takes place, as a rule of thumb, at an increasing price and technical complexity.

**Figure 6:**
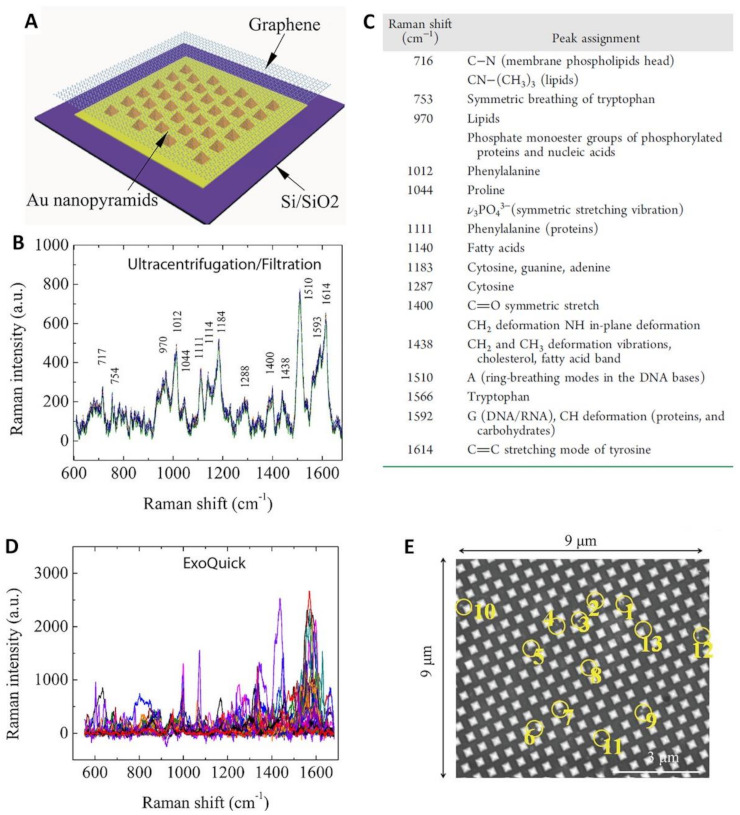
(**A**) Outline of the hybrid Au/graphene platform. (**B**,**D**) SERS spectra of exosomes isolated from fetal bovine serum using ultracentrifugation/filtration or the ExoQuick kit, respectively. (**C**) Assignment of the main SERS bands in (**B**). (**E**) A representative SEM micrograph of exosomes (circled in yellow) deposited onto the graphene-covered surface. Adapted with permission from [78]. Copyright 2019, American Chemical Society.

In this regard, Xie and co-workers [78] fabricated a substrate comprising a single-layer graphene overlaid on a periodic Au-pyramid nanostructure (Figure 6A). The graphene layer imparts a biocompatible and chemically stable surface while further boosting the amplification of the Raman signal via a chemical mechanism up to ca. 2 orders of magnitude [51,111]. In the same work, the authors highlighted the necessity of an efficient isolation procedure to enable a reliable exosome SERS fingerprinting analysis [78]. Exosomes from fetal bovine serum were isolated either via ultracentrifugation/filtration method or salting-out procedure using a commercial ExoQuick kit (System Biosciences LLC, Palo Alto, CA, USA). The former approach has the advantage of yielding purer samples while the second method is faster and capable of collect almost 1000 times more biomaterial but at the expenses of a lower purity. Particle size analysis of the two processed samples showed a similar mean diameter (ca. 135–143 nm range) but a narrower distribution for particles recovered by ultracentrifugation/filtration. Conversely, the outcome of the SERS analysis revealed many striking differences. Two μL of the exosome solutions were applied onto a hybrid plasmonic platform surface and allowed to air-dry before the measurement. A hundred of SERS spectra were collected on different spots of the platform, yielding reproducible fingerprint signatures for exosomes separated via ultracentrifugation/filtration (Figure 6B, see band assignment in Figure 6C) while ExoQuick-derived materials produced an ensemble of highly heterogenous vibrational profiles, preventing the acquisition of a unique and recognizable SERS spectrum (Figure 6D). The validity of the SERS platform for discriminating different populations of exosomes was demonstrated in combination with principal component analysis (PCA), using vesicles from different sources (fetal bovine serum vs human serum; and lung cancer cell lines HCC827 vs H1975). Interestingly, the authors also performed a dilution study to assess the possibility of performing single exosome analysis. SEM imaging was performed to visualize and count the exosomes localized over a specific area (Figure 6E). The so-estimated exosome density was correlated with the SERS mapping carried out on the same area (overlapping of adjacent laser spots for each SERS measurement was avoided). The results show a linear response of the overall SERS intensity with the change of the sample concentration, indirectly suggesting that individual SERS measurements possibly arise from the interrogation of single exosomes. In a separate work, Pramanik et al. [85] focused on maximizing the SERS response of a hybrid graphene-plasmonic substrate by embedding gold nanostars, one of most SERS efficient individual nanoparticles [55], into 2D graphene oxide structures. This hybrid substrate was successfully employed in the fingerprint identification and discrimination of exosomes derived from triple-negative breast cancer and HER2(+) breast cancer down to ca. 4 × 10^2^ exosomes/mL.

**Figure 7:**
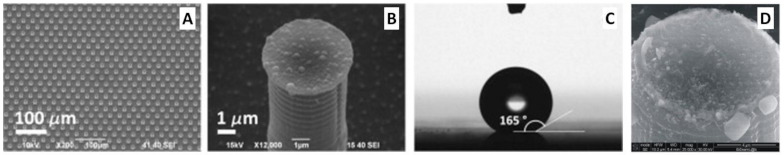
(**A**) A superhydrophobic surface consisting of periodic hexagonal patterns of cylindrical pillars. (**B**) A silicon micropillar with a randomly distributed silver nanograins. (**C**) A drop on top of the superhydrophobic surface displaying a contact angle as large as 165°. (**D**) Top view SEM image of exosomes on pillars. Adapted with permission from ref. [77]. Copyright 2012, Elsevier.

In addition to the intrinsic qualities of the plasmonic substrate, practical issues associated with the sample preparation can significantly impact the overall sensitivity of the method, such as the capability of concentrating vesicles in highly localized and electromagnetically active spots of the substrate. For instance, drop-casting of exosome dispersion onto a surface is typically affected by the coffee-ring effect, leading to an uneven distribution of the vesicles over a relatively broad area. Technically, SERS mapping of large areas (e.g., in the upper micrometric ranges) with high spectral resolutions to maximize the collection of intense signals is feasible but is typically a rather time-consuming process unless state-of-the-art techniques (e.g., SERS holography) are used [112]. A convenient way to concentrate diluted solutions of biological samples onto a small area is integrating plasmonic features on micro- and nano-patterned surfaces with superhydrophobic properties [113]. Superhydrophobic substrates typically comprises micro- and nano-textured surface imparting superior non-adhesive properties via entrapment of air pockets underneath a liquid droplet deposited on top of it. Thus, a droplet retains a quasi-spherical shape during evaporation rather than spread all over the surface, which progressively minimizes the contact area and, in turn, concentrates the analytes [114] over a small spot (less than few microns) [77]. Di Fabrizio and co-workers pioneered such an approach for the direct SERS analysis of exosomes [77]. In their work, a superhydrophobic array of silicon micropillars decorated with silver nanostructures (Figure 7A,B) was designed to discriminate exosomes isolated from either healthy (CCD841-CoN) or tumor (HCT116) colon cells using a commercial ExoQuick kit. Small drops of exosome dispersions (~0.2 ng/mL) were deposited on the substrates (Figure 7C) and, through evaporation, the vesicles were conveyed into small plasmonic-active regions of the substrate (Figure 7D) for the acquisition of averaged SERS spectra (50 acquisitions for each sample). More recently, Suarasan et al. [74] reported a simple, cheaper superhydrophobic plasmonic platform for SERS interrogation of exosomes in small sample volume (as low as 0.5 μL). A PDMS substrate consisting of nano- and micro-bowl structures exhibiting superhydrophobic properties was fabricated via soft lithography. Silver nanoparticles were then grown in situ to impart SERS enhancing properties.

**Figure 8:**
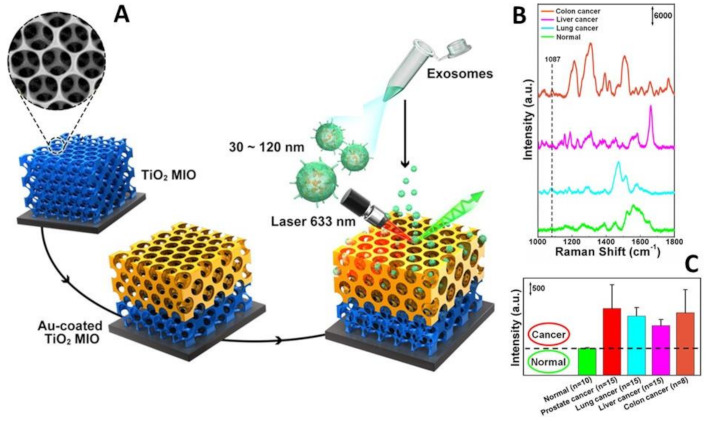
(**A**) Outline of the 3D gold-coated TiO_2_ macroporous inverse opal structure. (**B**) Typical SERS spectra of exosomes separated from plasma of normal individual and lung, liver, and colon cancer patients. (**C**) Averaged SERS intensity at 1087 cm^−1^ from exosomes separated from normal individuals and 15 lung cancer, 15 liver cancer patients and 8 colon cancer patients. The black dashed line shows the intensity boundary of the 1087 cm^−1^ peak between normal individuals and cancer patients. Adapted with permission from ref. [70]. Copyright 2020, American Chemical Society.

Alternatively, local enrichment of exosomes can be achieved via intracavity trapping. For instance, Xiao and co-workers [70] engineered a multifunctional 3D gold-coated TiO_2_ macroporous inverse opal structure (Figure 8A) providing (i) an interconnected beehive-like pore networks for trapping exosomes to improve their separation from the medium; and (ii) enhanced signal amplification within the cavity volumes as compared to flat or non-cavity structures. This latter effect results from the superimposition of the field enhancements from both the dipole resonance of the spherical cavity, which amplifies the intensity of the normal Raman signal of exosomes, and the strong plasmonic resonances at the gold film surface, enabling the corresponding SERS magnification under a 633 nm excitation. As a result, these hybrid structures appear particularly suitable for the interrogation of molecular objects in the exosome size-range. The authors exploited these materials for discriminating exosomes from healthy donors and patients diagnosed with lung, liver and colon cancer using the intensity of the 1087 cm^−1^ band as the spectral biomarker. This feature has been ascribed to the vibration of the P-O bond in the phosphate groups of phosphoproteins, which have been described as a protein biomarker of breast cancer-derived exosomes [115]. Exosomes were isolated from peripheral blood samples of cancer patients, using the Total Exosome Isolation Reagent (Invitrogen, Carlsbad, CA, USA) and dispersed in deionized water. 50 μL were then dropped onto the substrate (25 mm × 25 mm) substrate and dried naturally. SERS mapping measurements were finally performed over an area of 16.5 μm × 11.5 μm at 0.4 μm intervals to yield the resulting average spectra (Figure 8B). The intensity of the 1087 cm^−1^ SERS peak from the exosomes secreted by most of these lung, liver, and colon cancer patients was at least two times of that from healthy individuals (Figure 8C) while displaying much larger intensity fluctuations. The validity of the 1087 cm^−1^ band intensity as a spectral biomarker was further corroborated by the analysis of exosomes from the prostate, lung, liver, colon cancer cell lines.

As previously exploited for promoting the adhesion of plasmonic colloids in suspension onto exosomes, electrostatic interactions of the vesicles onto solid supports can also be employed to promote their local accumulation. For instance, Carney and co-workers [73] described the fabrication of a simple, low-cost plasmonic material comprising a microscale biosilicate material decorated with silver nanoparticles for SERS analysis of ovarian and endometrial cancer exosomes. Metallic surfaces were functionalized with cysteamine to impart positive charge and, therefore, favor the accumulation of exosomes in suspension via electrostatic binding with their negatively charged outer shell. Exosomes were initially isolated via differential ultracentrifugation from serum of 8 patients (6 of them with different cancer subtypes) and resuspended in up to 100 μL of ultrapure water. The samples were diluted 1:100 in pH 6.4 buffer and 30 μL drops were pipetted onto 2 mm × 5 mm substrate elements. Pretreatment of the substrates with the slightly acidic buffer was also performed to maximize the protonation of the cysteamine amino groups onto the silver surface. Upon incubation, SERS spectra were acquired in liquid condition on 5–10 different random spatial locations of the biosilicate plasmonic platform. Multivariate data analysis was successfully applied to distinguish tumor samples from healthy ones in patients suspected of gynecologic malignancy. A limit of detection (LOD) of less than 600 vesicles/mL has been reported.

**Figure 9:**
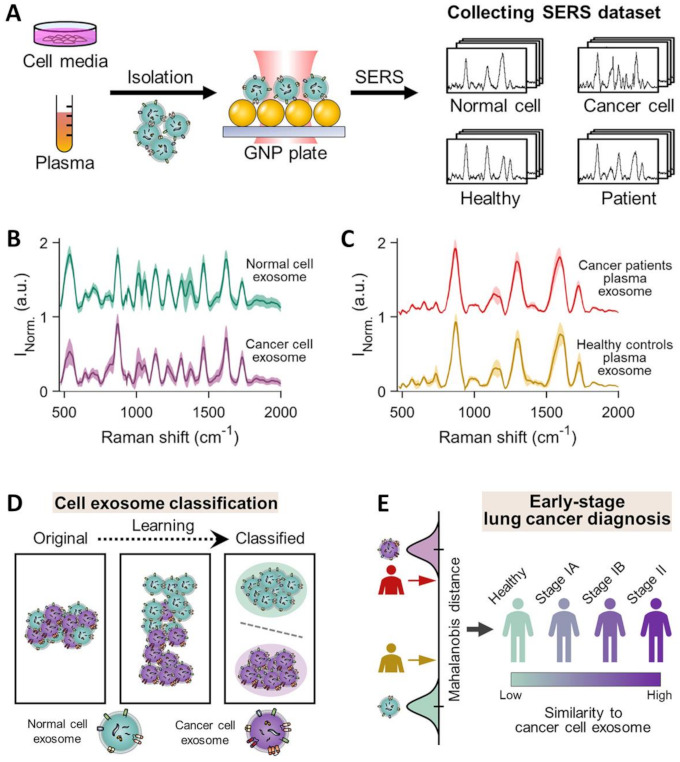
(**A**) Schematic of the collection of SERS spectra for exosomes isolated from different cell media and human plasma and dry-cast onto a gold-nanoparticle (100 nm) coated cover-slip. Specifically, cell-derived exosomes were isolated from lung-related cells: normal cell exosomes from human pulmonary alveolar epithelial cells (HPAEpiC) and cancer cell exosomes from A549, H460, H1299, H1763, and PC9 cells. Human plasma samples were collected from 20 healthy controls and 43 lung adenocarcinoma patients (22 patients in stage IA, 16 in stage IB, and 5 in stage IIB). (**B**,**C**) Average SERS signals of cell media supernatant-derived and human plasma-derived exosomes, respectively. (**D**,**E**) Overview of deep learning-based cell exosome classification and lung cancer diagnosis, respectively, using exosomal SERS signal patterns. Adapted with permission from ref. [90]. Copyright 2020, American Chemical Society.

Overall, direct SERS analysis in combination with multivariate statistical methods has fully demonstrated the consistent ability to discriminate exosomes isolated from different cell types. However, the complexity of exosome populations secreted from heterogeneous sources, such as those isolated from human blood, has significantly limited the viability of this approach as a diagnostic tool. As a striking example, Shin et al. [90] acquired the SERS spectra of cell-derived exosomes from healthy and cancer lung cell lines as well as human plasma exosomes from healthy controls and patients with different stages of lung cancer (Figure 9A). The average size of the examined exosomes fractions was similar (specifically, cell-derived exosomes = 139.6 ± 14.4 nm, human plasma-derived exosomes = 136.3 ± 3.2 nm). SERS measurements were performed by dry-cast exosome solutions onto gold nanoparticle decorated coverslips. Figure 9B illustrates the averaged SERS signals for normal cells and lung cancer cell exosomes which can be efficiently discriminated even by visual analysis. On the contrary, spectral differences between exosomes from plasma of healthy controls and lung cancer patients are negligible (Figure 9C), most likely due to the presence of a large number of exosomes from various organs that conceals the specific vibrational patterns of lung-derived exosomes. To overcome this limitation, the authors employed deep learning algorithms to analyze the spectroscopic signals of exosomes. Deep learning (DL) is a machine learning method based on artificial neural networks that effectively process big sensing data for complex matrices or samples, allowing classification, identification, and pattern recognition [116]. Notably, DL algorithms have shown to be extremely beneficial for analyzing spectroscopic data in biosensing applications [116]. In this abovementioned work [90], besides a mere classification of the SERS data from healthy controls and cancer patients, deep learning was used to establish a correlation between the exosome data from individual lung cells with the overall patient’s histological characteristics. Specifically, the spectral data set of cell-derived exosomes were first used to train the DL models for binary classification of cell types which, subsequently, efficiently separated exosomes from human plasma (healthy vs cancer) into two clusters with an accuracy of 95% (Figure 9D). Finally, using PCA scores at the terminal fully connected layer, the Mahalanobis distance between plasma and cell exosome clusters is determined to quantitatively evaluate the resemblance of the data from plasma and cancer cell exosomes. The DL model predicted that 90.7% of plasma exosomes from 43 patients, including stage I and II cancer patients, had higher similarity to exosomes derived from lung cancer cell lines than the average of the healthy controls (Figure 9E). Remarkably, the degree of such similarity correlates to the progression of cancer. It is worth stressing that, besides reducing the need of acquiring a sufficiently large number of patient samples to generate a robust and reliable data set for discrimination, this approach provides the biological basis for classification. This reduces the impact of undetected potential experimental errors as the source of spectral differences.

**Figure 10:**
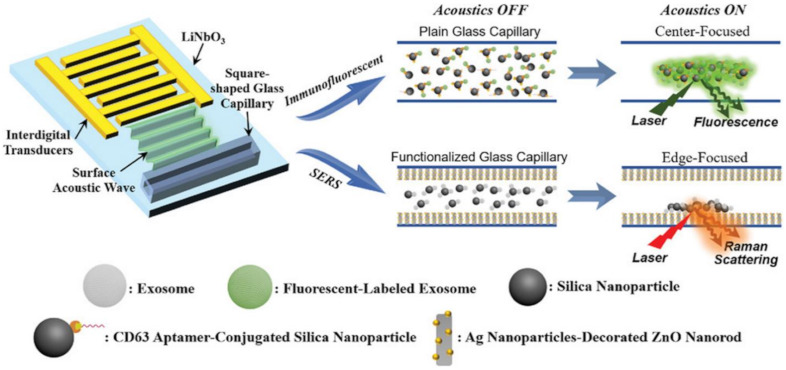
Outline of the acoustofluidic biosensor features and mechanisms of optical detections (immunofluorescence and SERS sensing). The device comprises a transparent piezoelectric lithium niobate (LiNbO_3_) substrate with patterned interdigital transducers (IDTs) and a square-shaped glass capillary bonded to the substrate. Surface acoustic waves concentrate particles at either the center or the perimeter of a glass capillary. Adapted with permission from [89]. Copyright 2020, Wiley-VCH.

Integration of SERS sensing and Raman components into multifunctional platforms with additional features (e.g., microfluidics and magnetic separation for sample handling, fluorescence spectroscopy for multimodal optical analysis, etc.) [62,117,118] has been intensely pursued in recent years to overcome intrinsic limitations of SERS as a stand-alone technique and, thus, pave the way for the fabrication of miniaturized biosensor for point-of-care testing. In particular, Raman spectroscopy can be easily combined with microfluidics, a technology that facilitates high throughput and automated analysis with very low sample consumption [62,117]. In exosome analysis, Hao et al. [89] combined acoustics and microfluidics technologies with dual fluorescence and SERS optical detection into a single analytical device. The main features of the fabricated acoustofluidic platform are outlined in Figure 10. The device generates surface acoustic waves (SAWs) that propagate toward the glass capillary microchannel where the exosome suspension is confined. This results in pressure fluctuations within the liquid that force the suspended particles to concentrate at the center of the fluid chamber (for immunofluorescent detection) or the edge near a plasmonic Ag nanoparticle-deposited ZnO nanorod arrays (for SERS analysis). To this end, CD63 aptamer-conjugated 400 nm silica nanoparticles were used to capture exosomes and enable their acoustic-assisted enrichment at the plasmonic substrates for highly sensitive label-free SERS detection down to ca. 20 exosomes per μL (from human plasma-derived exosome samples).

## 4. Indirect SERS Analysis of Exosomes Using SERS-Encoded Nanoparticles (SERS tags)

**Figure 11:**
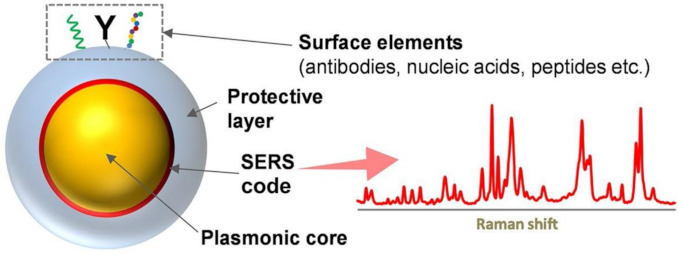
Depiction of a representative example of SERS-encoded particle (SEP) or SERS tag. Adapted with permission from [119]. Copyright 2017, Springer Nature.

A large variety of SERS-encoded particles (SEPs), or also referred to as SERS tags, with different structural and chemical features has been reported in the literature [68,119,120,121]. Despite a broad range of diversity, it is possible to recognize the following key building units (Figure 11): (i) a nanoparticle-based core (typically, silver or gold) as the plasmonic enhancer, (ii) a dense collection of molecules with large Raman cross-sections (referred to as codes or labels or reporters) attached to the metallic surface to provide an intense and well-defined vibrational fingerprint, and (iii) a variety of surface molecular ligands (e.g., antibodies, aptamers, peptides) to impart selectivity toward a target analyte. These recognition elements are often conjugated onto the surface of a protective inert layer (e.g., silica) coating the SERS labelled plasmonic core, which is integrated into the nanomaterial to afford high stability in complex media and avoid leaking of the codes [119]. Indirect sensing with SEPs is entailed with multiplexing capabilities with single laser excitation, thanks to the unique vibrational fingerprints of each code, and quantitative response, as the SEP structure can be engineered to provide a SERS intensity that would scales linearly with the SEP content [119].

**Figure 12:**
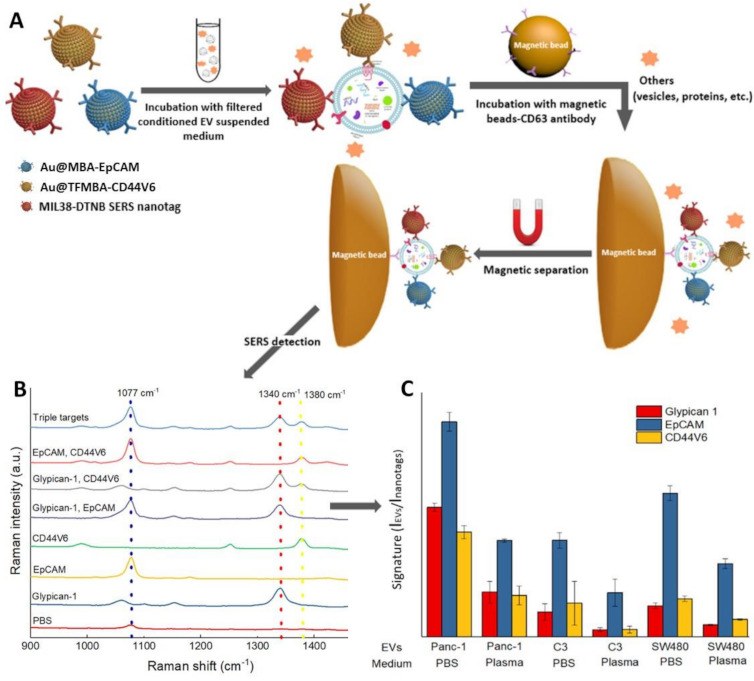
(**A**) Schematic illustration of molecular phenotype profiling of CD63-positive exosomes using CD63 antibody-functionalized magnetic beads and three classes of SERS-encoded nanoparticles separately conjugated with antibodies targeting Glypican-1, EpCAM, and CD44V6 surface biomarkers. SERS codes: 5,5′-dithiobis(2-nitrobenzoic acid) (DTNB); 4-mercaptobenzoic acid (MBA), and 2,3,5,6-Tetrafluoro-4-mercaptobenzonic acid (TFMBA). (**B**) SERS spectra for the simultaneous detection of three biomarkers on Panc-1-derived exosomes in PBS. Peaks at ca. 1077, 1340, and 1380 cm^−1^ are correlated with the presence of EpCAM, Glypican-1, and CD44V6, respectively. (**C**) Phenotypic signature of Panc-1-, C3-, and SW480-derived exosomes in PBS and plasma (n = 3). Adapted with permission from [97]. Copyright 2020, American Chemical Society.

In the indirect SERS analysis of exosomes, SEPs are conjugated with recognition elements that promote their selective accumulation at the surfaces of the vesicles. To facilitate the SERS interrogation, capturing substrates are also integrated into the sensing system to enable the separation and accumulation of the SEPs-decorated exosomes into a small area for ultrasensitive detection. Typically, capturing substrates consist of magnetic beads [92,93,94,96,97,122] or flat supports [98,99,100] functionalized with further recognition molecules for specific exosome binding. Notably, such an approach also removes the need for time-consuming, costly and complex exosome isolation procedures (e.g., ultracentrifugation) as SEPs and capturing substrates can be directly applied to biological media (e.g., conditioned medium, serum, blood, urine, saliva) for exosome binding and separation. The multiplexing capability of indirect SEP-based sensing makes this method particularly suited for phenotypic profiling of transmembrane proteins of cancer-derived exosomes [93,97,99]. Simultaneous evaluation of the expression levels of multiple surface proteins (phenotype) provides a much more reliable molecular description of the heterogeneous nature of tumour-derived exosomes as compared to a single marker characterization [97]. Notably, multidimensional phenotyping has shown to play a central role in improving diagnostic, drug treatment, disease monitoring and prognosis [47,123]. In a proof of concept study, Wang and co-workers [97] demonstrated the viability of such an approach by profiling three surface biomarkers of pancreatic cancer (Glypican-1, epithelial cell adhesion molecules—EpCAMs, and CD44 variant isoform 6—CD44V6) on exosomes secreted by a human pancreatic cancer cell line (Panc-1). Exosomes of ca. 130 nm size suspended in a conditioned medium were obtained upon removing cells from the culturing media. Aliquots of conditioned exosomes were either diluted in either PBS or plasma from healthy subjects and, subsequently, directly combined with an equimolar mixture of three classes of SEPs (Figure 12A). The different batches of SEPs (Au@MBA-EpCAM; Au@TFMBA-CD44V6 and Au@DTNB-MIL38) comprise 55 nm gold nanoparticles labelled with a unique molecular code (DTNB, MBA or TFMBA) and conjugated with one CD44V6, EpCAM and MIL38 monoclonal antibodies (MIL38 is specific to Glypican-1). The mixture was stirred for 1 h before adding exosome-specific CD63-modified magnetic beads to enable, after an additional hour of incubation, the magnetically-assisted separation of the sandwich-like immunocomplex (Figure 12A). An enriched immunocomplex suspension in PBS was then interrogated by SERS, providing highly averaged spectra (Figure 12B) where the intensities of marker bands of the three codes (1077, 1340 and 1380 cm^−1^ for MBA, DTNB and TFMBA, respectively) indirectly inform about the relative expression levels of surface protein biomarkers. The phenotype signature was expressed by normalizing the corresponding Raman signals (I_EVs_) and SEPs (I_nanotags_) using the same concentration of conditioned exosomes. The study was also repeated with C3 (bladder cancer) and SW480 (colorectal cancer)-derived exosomes. Figure 12C illustrates the corresponding phenotypic signatures in either PBS or exosome-spiked plasma from healthy people. The results display a different degree of both absolute and relative surface proteins levels, with EpCAM the more expressed one. This is consistent with the current knowledge of EpCAM as the most highly expressed cancer biomarker. Molecular profiles show high similarity in both PBS and plasma suspended exosomes, but with a consistently lower absolute intensity for exosome dispersed in the plasma medium. This has been tentatively ascribed to a dilution of the cancer-specific signals due to the presence of additional exosomes equipped with surface CD63 antigens that are also magnetically separated by the CD63-conjugated magnetic beads. The sensitivity of the assay was determined to be 2.3 × 10^6^ particles/mL in PBS, which is below the average exosome concentration in most body fluids (ca. 10^8^ EVs/mL and above), thereby meeting the clinical requirements.

**Figure 13:**
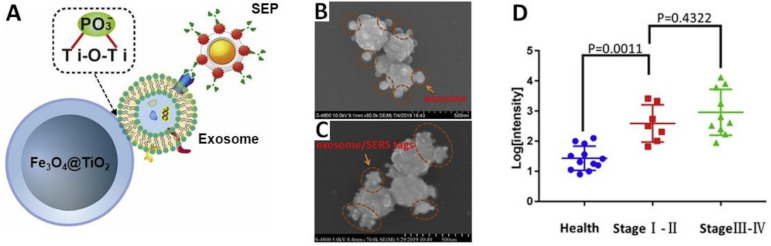
(**A**) Outline of the sandwich complex between TiO_2_-coated Fe_3_O_4_ beads (Fe_3_O_4_@TiO_2_), exosomes and SERS-encoded nanoparticles (SEPs) comprising a gold-silver core-shell nucleus functionalized with 4-mercaptobenzoic acid (MBA) as the SERS code and further conjugated with an anti-PD-L1 antibody. (**B**,**C**) SEM images of Fe_3_O_4_@TiO_2_ + A549 exosome, and Fe_3_O_4_@TiO_2_ + A549 exosome + SEPs, respectively.(**D**) Scatter plots of the log SERS intensity (MBA band at ca. 1074 cm^−1^) in the serum samples from the controls and the early-stage (stage I/II) and advanced (stage III/IV) patients diagnosed with non-small-cell lung carcinoma (NSCLC). Adapted with permission from [95]. Copyright 2020, Elsevier.

The use of immunoaffinity magnetic beads for separation/enrichments of exosomes from biofluids is, however, often affected by issues related to limited reproducibility, long incubation time and low exosome yields (<50%) [95]. On the other hand, the use of capturing substrates with one, highly specific recognition element such as an antibody, can introduce biases in the exosome isolation, leading to the enrichment of distinct exosome subpopulations to the detriment of others which, however, may be critical to diagnosis [124]. To this end, Pang et al. [95] replaced immunoaffinity beads with TiO_2_-coated magnetic particles, which allowed the indiscriminate, rapid and highly efficient removal of the exosomes from serum by exploiting the affinity of TiO_2_ for binding the hydrophilic phosphate head of the exosomal phospholipids. In their study, the programmed death ligand 1 (PD-L1) protein biomarker on the exosomal membrane was subsequently targeted with SEPs modified with an anti-PD-L1 antibody (Figure 13A). Exosomes derived from adenocarcinoma human alveolar basal epithelial cells (A549) were initially used as model samples because of their PD-L1 expression level closely correlated with lung cancer stage. Figure 13B,C show SEM images that visualize the capturing of the exosomes onto the Fe_3_O_4_@TiO_2_ particles and the subsequent binding of SEPs onto the exosomal surfaces. Laser interrogation of the aggregates yields SERS intensities that linearly scales with the exosome concentration in the 5 × 10^3^ to 2 × 10^5^ particles/mL range, with a detection limit of 1 PD-L1 + exosome/μL and an exosomal capture efficiency of 96.5%. The assay was finally tested with human serum samples from healthy donors (12) and NSCLC patients of early (7) and advanced stages (10). Figure 13D shows the scatter plots of the log SERS intensity for each group of samples. Clear separation can be observed for the healthy persons and the diagnosed patients, whereas discrimination has not been successfully achieved for this cohort of stage I-II and stage III-IV patients. Notably, this strategy allows the ultracentrifugation-free quantification of exosomal PD-L1 by using only 4 μL clinic serum sample and in less than 40 min in total (much lower than the 2–5 h time reported by other exosomes detection methods) [95].

**Figure 14:**
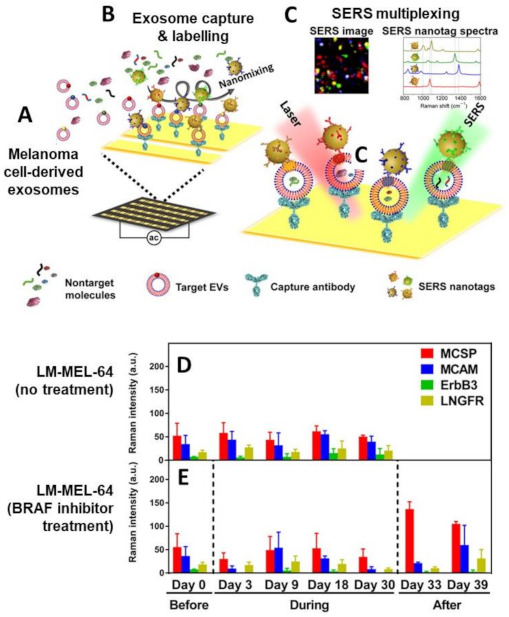
(**A**–**C**) Schematic of the exosome phenotyping using SERS-encoded nanoparticles: (**A**) Exosomes are secreted by melanoma cells with a BRAF V600E mutation in the culture medium or into circulation; (**B**) The exosome containing sample is injected, together with SERS tags, into the nanomixing chip equipped with capture antibodies; (**C**) Upon removal of non-target molecules (e.g., protein aggregates and apoptotic bodies) and unbound SERS tags, SERS mapping is performed to provide the SERS phenotyping of the captured exosomes. The false-color SERS image is generated from the characteristic peak intensities of each SERS tags (MCSP-MBA, red; MCAM-TFMBA, blue; ErbB3-DTNB, green; LNGFR-MPY, yellow). (**D**,**E**) Phenotypic alterations of exosomes derived from melanoma patient-derived LM-MEL-64 cell line in response to BRAF inhibitor treatment at different times (before, during and after treatment). Anti-CD63 antibodies were used in the capturing area. Adapted with permission from [99]. © The Authors, some rights reserved; exclusive licensee AAAS. Distributed under a Creative Commons Attribution NonCommercial License 4.0 (CC BY-NC). Available online: http://creativecommons.org/licenses/by-nc/4.0/ (accessed on 30 April 2021).

Alternatively, Trau and co-workers [99] tackled the limitations of immune-affinity separation and slow binding kinetics by integrating a nanomixing strategy that improves exosome capture efficiency while reducing non-specific adsorption and incubation time. A chip implementing nanomixing forces was designed for the streamlined plasma exosome phenotype analysis in less than 40 min, as outlined in Figure 14A–C. Exosomes derived from melanoma cell lines of patients treated with the BRAF inhibitor were selected to evaluate responses to the treatment. BRAF inhibitor targets BRAF V600, a mutation found in ca. 40% of melanoma patients that promotes cell cycle progression and tumor growth. The cell culture medium or diluted patient plasma containing the exosomes are directly fed into the capturing chip without any previous purification and enrichment steps (Figure 14A). The capturing area was modified with an anti-CD63 antibody targeting a generic, non-cancer specific exosome biomarker to maximize the vesicle accumulation at the interrogation spot. Exosomes are then simultaneously targeted by a pool of four classes of SEPs (Figure 14B), which comprise gold nanoparticles labelled with unique SERS codes and tumour-specific antibodies targeting four biomarkers that have been previously shown to undergo changes in expression levels with treatment and melanoma progression (i.e., melanoma chondroitin sulfate proteoglycan—MCSP, melanoma cell adhesion molecule—MCAM, low-affinity nerve growth factor receptor—LNGFR, and receptor tyrosine protein kinase—ErbB3). SERS mapping of the surface capturing area (Figure 14C) collects spectral intensities that are proportional to the numbers of exosomes and their expressing biomarker levels. Thus, as previously discussed, the exosome phenotype can be extracted by determining the relative SERS intensities of the code marker bands. Characterization of the phenotypic changes during treatment was first demonstrated on exosomes from patient-derived melanoma cell lines harbouring either a BRAF mutation (e.g., LM-MEL-64) or an NRAS mutation in a BRAF wild type (experimental control). For instance, exosomes collected from LM-MEL-64 cells without drug treatment did not show any significant changes across four selected biomarkers (Figure 14D). Upon drug exposure, however, it is visible a radical reshaping of the protein expression levels followed by a general up-regulation of the MCSP and MCAM levels once the BRAF inhibitor treatment was interrupted (Figure 14E). It is worth noting that, when anti-CD63 antibody at the capturing area was replaced with anti-MCSP, exosomes cell-derived phenotypes (specifically, from SK-MEL-28 cell lines) were different, suggesting heterogeneity of secreted vesicle subpopulations. Such heterogeneity further reflects a potential genetic or epigenetic variability within the cell population. The method was finally validated by monitoring the evolution of cancer-specific exosomes phenotypes from the plasma of melanoma patients receiving targeted therapy, which reflects the potential of exosome phenotyping for monitoring treatment responses.

Increasing the density of biologically functioning antibodies at the capturing surface is also a central factor for improving the efficiency and sensitivity of the immunoassay. In this regard, Li et al. [100] reported the use of polydopamine (PDA) self-polymerizing on glass slides to generated a 50–100 nm thick, rough layer suitable for enhanced antibody anchoring. Similarly, SEPs were fabricated with a thin PDA shell for antibody conjugation. Overall, the PDA encapsulation yields a more uniform, mild and biocompatible surface functionalization which entails high antibody capture efficiency and high sensitivity for detecting cancer-derived exosomes. PDA technology was integrated into a miniaturized device for the monoplex SERS analysis of 2 μL samples from clinical serum collected from healthy donors and pancreatic cancer patients. The assay efficiently discriminated between healthy donors and patients as well as between patients with different stage tumors (discriminatory sensitivity = 95.7%). Also, the assay displayed high sensitivity with a detection limit estimated to be just one single exosome per 2 μL for cell line-derived samples.

## 5. Future Challenges

In this review, we summarized and coherently discussed the diverse applications of SERS in the analysis of exosomes, with a special focus on the more recent and promising advances. We have also progressively highlighted current key challenges and limitations, which can be broadly associated with either the general application of SERS in biosensing and clinical diagnostic or the specific nature of exosomes as the biological target. In the first case, the translation of SERS-based analytical tools into competitive, commercial devices still faces important practical obstacles such as the production of cost-effective, robust and efficient plasmonic substrates at a large scale. Similarly, the fabrication of affordable and portable Raman spectrometers for fast data acquisition is critical for lowering the cost while providing manageable equipment for routine analysis in the clinical setting. In this regard, the integration of SERS substrates and Raman components into multifunctional platforms (e.g., microfluidics) is also pivotal for automatization and efficient standardization of the measuring procedures. Furthermore, as also stressed in the review, the efficient implementation of the most advanced chemometric tools appears to be the way to fully access the multidimensional information contained in large SERS data set. On the other hand, the intrinsic nature of these vesicles makes exosome-based diagnostics a difficult task, mainly due to their pronounced molecular heterogeneity and the requirement of determining the presence and relative distribution of different sub-populations, especially in complex biofluids. As pointed out, both improvement and standardization of the isolation procedures are needed to reproducibly supply exosomes with high purity in good yields, while identification of a much broader set of potential biomarkers is mandatory for enabling clinical applications.

## Data Availability

Not applicable.
